# Perinatal healthcare access, perceived quality, and preferences among historically underrepresented mother–infant dyads: a mixed methods study

**DOI:** 10.1186/s12884-025-08029-6

**Published:** 2025-08-26

**Authors:** Nicole C. Amodio, Victoria Cueto, Elizabeth Znamierowski, Tabassum Firoz, Sarah N. Taylor, Ruthfirst E. A. Ayande, Leslie Sude, Julia Rosenberg

**Affiliations:** 1 Yale School of Medicine, Yale University, New Haven, CT USA; 2 Department of Pediatrics, Yale University School of Medicine, Yale University, New Haven, CT USA; 3 Yale University, Infectious Diseases, New Haven, CT USA; 4 Department of Obstetrics, Gynecology, and Reproductive Sciences, Yale University, New Haven, CT USA; 5 Bridgeport Hospital, Bridgeport, CT USA

**Keywords:** Pregnancy, Maternity care, Postpartum, Social support, Health education, Empowerment, Dyadic care

## Abstract

**Background:**

Timely and accessible prenatal and postpartum healthcare supports the health and well-being of the mother–infant dyad, enabling detection and prevention of pregnancy-related complications and chronic conditions. In the U.S., maternal mortality and morbidity are disproportionately higher in Black and Hispanic/Latina women. Focused, intentional dyadic care informed by patient perspectives can improve healthcare access and mitigate inequities.

**Aim:**

To understand factors influencing access to and perceived effectiveness of perinatal healthcare for mother–infant dyads by exploring perspectives, preferences, perinatal health knowledge, and opportunities to empower mothers from historically marginalized or underrepresented backgrounds, to promote health advocacy.

**Methods:**

In this mixed-methods study, our interdisciplinary team administered an iteratively developed interview guide and survey with purposively sampled English/Spanish-speaking women who received care at a maternal-infant mobile medical clinic or a postpartum heart health program, settings where patients are primarily covered by Medicaid insurance. Phone interviews were recorded via a videoconferencing app, transcribed with automated software, and edited by research team members. A four-person coding team (two of whom speak Spanish) coded transcripts in original languages in Dedoose. Discrepancies were resolved by consensus, and the constant comparative method was applied to identify emerging themes. Descriptive statistics were applied to quantitative data, which consisted of a brief survey of participant demographics, health problems, and preferences for perinatal care.

**Results:**

Of the 22 in-depth interviews (9 Spanish,13 English), half chose either a home or mobile medical clinic visit as their preferred care setting, and half reported a missed clinic-based appointment due to lack of transportation. Key themes reflected postpartum healthcare priorities: (1) multi-level support centered around mother–infant dyad preference (cross-cutting theme); (2) collective well-being (fostering connection and communal experience); (3) improved provider communication and education (to empower self-care and health knowledge); and (4) responsive and personalized perinatal care (meeting women where they are at).

**Conclusion:**

Women’s perspectives reflected the need for multilevel support with specific attention to community and relationship strengthening, provider engagement, and policy reforms around social support (e.g., benefits and insurance). Findings suggested that prioritizing holistic, patient-centered perspectives may enhance interactions with healthcare systems and contribute to improving perinatal health equity.

**Supplementary Information:**

The online version contains supplementary material available at 10.1186/s12884-025-08029-6.

## Background

Recent data show that U.S. maternal mortality rates rank the highest compared with 13 other high-income countries, with mortality rates sometimes double or triple that of peer nations [1]. In 2021, the US had 33.2 pregnancy-related deaths per 100,000 live births overall, with significant racial and ethnic disparities [2]. Pregnancy-related deaths are defined by the Centers for Disease Control and Prevention as deaths during or within one year of the end of pregnancy from any cause related to or aggravated by pregnancy. Non-Hispanic White women had 24.3 pregnancy-related deaths per 100,000 live births, compared with 31.4 for Hispanic/Latina women and 69.3 for non-Hispanic/non-Latina Black women [2]. While there are opportunities for improvements through all perinatal phases, the postpartum period is a critical touchpoint to mitigate pregnancy-related deaths as 63% of these deaths occur after delivery [3]. The postpartum period presents an opportunity to engage women in ongoing healthcare beyond the immediate peripartum period through the provision of health assessments to the mother–infant dyad [4, 5].

In 2018, the American College of Obstetrics and Gynecology (ACOG) sought to reduce maternal mortality and morbidity in the United States by redefining the recommendations for postpartum care [6]. The ACOG recommendations included an initial assessment within the first three weeks postpartum, ongoing tailored care as needed, and a full, comprehensive exam within the first 12 weeks postpartum [6]. However, only an estimated 72.1% of women attend a postpartum healthcare visit, and attendance rates dropped further during the COVID-19 pandemic, especially for women in racial and ethnic minority groups [7, 8]. Lower postpartum visit attendance is associated with uninsurance and underinsurance, mental health or substance use disorders, transportation barriers, housing instability, limited employment, and residence in high-poverty neighborhoods [9]. Experiences of discrimination and racism also contribute to lower postpartum visit attendance. A nationally representative survey found that mothers who perceived disrespect during childbirth were twice as likely not to return to postpartum care compared with their peers after adjusting for socioeconomic and medical characteristics [10]. While various factors contribute to decreased postpartum visit attendance, having an established relationship with a healthcare provider may promote continued care. A study of predominantly Black women found that having a personal connection with a provider who was familiar with them and their health history was significantly associated with attending postpartum visits [11]. Qualitative research further underscores the importance of patient-provider relationships and healthcare interactions, especially for those in historically underrepresented groups. A qualitative systematic review and meta-aggregation found that people who identified with historically marginalized racial and ethnic groups often experienced ineffective communication, a lack of cultural sensitivity, and discrimination in maternity care settings [12].

An opportunity to increase engagement in postpartum healthcare is through the provision of dyadic care, the concurrent provision of healthcare to both infant and mother. Integrating healthcare for both the woman and her child acknowledges the prevailing tendency of mothers to prioritize their children’s medical needs, sometimes at the expense of their own well-being [13, 14]. Mothers have expressed a desire for and interest in an integrated approach to care, where their healthcare is delivered in tandem with their infant’s care [15]. Implementing healthcare strategies adapted from existing dyadic models (e.g., family medicine, midwifery) can improve the integration and delivery of care for the dyad [16]. Researchers have found that integrating maternal care into children’s visits creates opportunities to screen for and address behavioral health risks, diabetes, mental health concerns, and social services needs and can increase postpartum visit attendance [17–20]. Moreover, dyadic models of care have resulted in greater utilization of primary care and behavioral health in younger, publicly insured mothers from historically marginalized racial and ethnic groups [20]. The COVID-19 pandemic underscored the need to reach mothers with health-related social needs and individuals facing significant healthcare barriers, spurring the implementation of new dyadic care in mobile medical clinics (MMCs) [4, 21]. Mother–infant MMCs offer a dyadic approach to improve access by bringing convenient health care to recently delivered mothers, which in turn can increase postpartum attendance and address unmet medical and social needs [22]. Yale’s Mother-Infant-Program, founded in 2020, is one example of a dyadic care MMC [4].

As dyadic models and MMCs continue to expand, understanding women’s care preferences can improve care delivery and promote postpartum healthcare. Recent studies have focused on care preferences related to perinatal telehealth visits and the COVID-19 pandemic [23, 24]. One study found that mothers expressed interest in more postpartum visits and were open to home visits; however, this analysis surveyed a largely white and privately insured patient population [25]. A 2014 study explored preferences among low-income women and found an openness to contraceptive provision during well baby visits and a desire for flexible postpartum visits, mental health resources, and earlier access to contraception [26]. This study was conducted before the COVID-19 pandemic and the ACOG’s change in recommendations for postpartum care.

To bridge the gap in understanding of care preferences among mothers from historically marginalized groups in the post-COVID era, we aimed to assess patient perspectives on postpartum health, health information delivery, and care settings. Our evaluation focused on individuals from a region with high poverty rates, populations with Medicaid or uninsurance, and historically underrepresented minority communities.

## Methods

### Study design

We employed a convergent mixed-methods study design to collect and analyze qualitative and quantitative data. Semi-structured telephone interviews were concurrently conducted with the administration of structured telephone surveys from March to June 2024. Participant age and gestational age at delivery were extracted from electronic health records following permission from participants after completion of the interview and survey.

### Setting

#### Study location

We conducted this study in catchment areas for academic institutions in the greater New Haven and Bridgeport regions of Connecticut, United States. Both Bridgeport and New Haven have diverse populations: Bridgeport’s population is 36% Hispanic/Latina and 20% Black, and New Haven’s is 31% Hispanic/Latina and 30% Black [27, 28]. In the greater New Haven area, 14% of adults experience transportation insecurity, and 13% face food insecurity [27]. Moreover, 12% of New Haven adults reported feeling discriminated against in medical settings within the past three years [27]. Notably, these regions have a Black infant mortality rate of 11.3 deaths per 1000 live births, compared to an overall infant mortality rate of 5.93 and 7.2 per 1000 live births for New Haven and Bridgeport, respectively [27, 28]. Additionally, 4.5% of Hispanic/Latina women in greater New Haven had late or no prenatal care, and 12.9% of Black women in Bridgeport gave birth to an infant with low birthweight, compared with 8.6% overall rate of low birthweight in the U.S [27–29].

#### Recruitment sites: mother–infant dyadic mobile medical clinic intervention and postpartum heart health clinic

The Yale Mother-Infant-Program delivers postpartum care to mother–infant dyads with health-related social needs or known maternal risk factors for postpartum complications. Since its inception, the Mother-Infant-Program has completed over 1900 visits, with services provided by an APRN, usually delivered directly outside family homes. Infant care includes biometric assessment (length, weight, head circumference), physical exams, and transcutaneous bilirubinometry. Maternal care includes screenings for maternal hypertension, preeclampsia, and postpartum depression; lactation assessments and guidance; same-day referrals to high-risk maternal fetal medicine or direct referral to the emergency department when required; and diaper and menstrual product distribution [30, 31].

The Postpartum Heart Health Clinic at Bridgeport Hospital in Connecticut provides a comprehensive assessment of cardiac risk for new mothers who had pregnancy complications that increase the risk of future heart disease (such as hypertensive disorders of pregnancy and gestational diabetes) by providing culturally contextualized and individualized plans for cardiac risk reduction.

### Participants

We sampled participants who were recently seen at both recruitment sites, where most patients identified with historically marginalized racial/ethnic groups in electronic health records and/or used Medicaid. From eligible participants, we purposively sampled those who spoke English or Spanish. Inclusion criteria were: [1] receipt of care at either Bridgeport Hospital’s Postpartum Heart Health Clinic or the Yale Mother-Infant-Program [2], recent (within the prior year) birth, and [3] English or Spanish as preferred language in their electronic health record. The study was deemed exempt by the Yale Institutional Review Board (#2000036626).

### Development of interview guide and survey

The interview guide and survey were iteratively developed by our research team to focus on constructs related to patient-centered care with the goal of improving perinatal care delivery from the patient perspective [32]. We used the following conceptual domains in designing our interview guide and survey: healthcare delivery and utilization, barriers to care, health knowledge and perspectives, primary care, perinatal physical and mental health, support systems, and traditional practices. The conceptual domains of interest were informed by thematic findings from previous evaluations of the Yale Mother-Infant-Program and a review of existing literature investigating current gaps in maternal preferences utilizing PubMed, PsycINFO, and Google Scholar databases [4, 23–26]. Since we defined the conceptual domains in advance, we designed qualitative questions in the interview guide and quantitative questions in the survey to be closely related and inform similar topics [33, 34]. By utilizing this mixed methodology “matching” strategy, we aimed to optimize holistic understandings of each domain of interest via subsequent integration of qualitative and quantitative findings [33, 34]. The survey included demographic questions modeled on the CDC’s National Health and Nutrition Examination Survey [35]. The full interview guide and survey are attached in Additional file 1.

### Quantitative and qualitative data collection

An English- or bilingual (English- and Spanish)-speaking research assistant (NA, VC) who had not previously worked with the eligible participants called via telephone to inquire about participation and schedule a time for informed consent and survey/interview. All participants provided verbal informed consent for the telephone interview/survey and for collection of pre-specified data from their health records. The research assistants conducted telephone interviews and surveys and separately recorded contact information to send all participants a $20 gift card. The interview and survey were audio-recorded using the health information compliant Zoom feature, transcribed using Microsoft Word, and manually edited by the interviewer for accuracy. Quantitative survey data and informed consent documentation were recorded and stored in Qualtrics^®^ (Qualtrics, Provo, Utah). Unique identifiers were generated for each participant without recording names in the Qualtrics platform. We stopped recruitment once thematic saturation of the interviews was reached [36]. The full interview and survey ranged from 23 to 63 minutes in duration.

### Mixed methods analyses

We followed the COnsolidated criteria for REporting Qualitative research (COREQ, Additional file 2) and Strengthening The Reporting of OBservational studies in Epidemiology (STROBE, Additional file 3) checklists to inform reporting of study design, data collection and analysis, and reporting of qualitative and quantitative data, respectively [37, 38]. We first analyzed transcripts deductively, applying codes related to categories from the interview guide, and then used an inductive approach to determine emerging themes via the constant comparative method in Dedoose^®^. A five-person, female coding team of two research assistants (NA, VC), two pediatric clinician scientists (JR, LS), and a nurse practitioner (EZ) independently created codes. Discrepancies in codes were resolved by consensus. The research team discussed all codes and grouped them to create a codebook and assess emerging themes.

Following our inductive thematic analysis, we applied the National Minority Health and Health Disparities (NIMHD) Research Framework to guide thematic re-categorization of qualitative findings and connect themes. The NIMHD framework provides a classification structure to understand the relevant factors for the complex, multi-faceted nature of minority health and health disparities [39]. The NIMHD framework lends itself as a vehicle to examine maternal domains of influence (biological, behavioral, physical/built environment, sociocultural environment, health care system) and different levels of influence (individual, interpersonal, community, societal) within those domains [39].

The integration of quantitative and qualitative data was achieved by matching, comparing, and expanding [34]. The matching strategy reflected the interview and survey design with pre-specified domains of interest in both qualitative and quantitative concurrent interview and survey findings [33, 34]. The qualitative and quantitative data were then analyzed in parallel, before comparing and contrasting to identify convergent and divergent findings between the interviews and surveys [33, 34]. We then employed the expanding strategy, leveraging the qualitative data to broaden and contextualize the findings of the quantitative data [34].

### Language considerations

Two members of the research team were proficient in Spanish, and we utilized a professional translation service to translate both the informed consent document and interview guide/survey into Spanish. The recruitment phone calls and telephone interviews/surveys were conducted in the participants’ preferred language. Coding of the qualitative data was completed in the original language of the interview, a methodology that can ensure cultural considerations which may be overlooked in translation are explored [40]. Translation of relevant Spanish quotations into English were completed by the study team for this publication, with original Spanish quotations in the Additional file 4.

#### Note on gendered language

We recognize that not all individuals assigned female at birth or those who experience pregnancy and childbirth identify as women. While we did not ask participants how they identified in relation to their pregnancy experience, those who took part in this study referred to themselves using she/her pronouns or described their experiences in ways typically associated with womanhood. In this paper, we use gendered terms in a way that aligns with how participants shared their experiences.

## Results

**Fig. 1:**
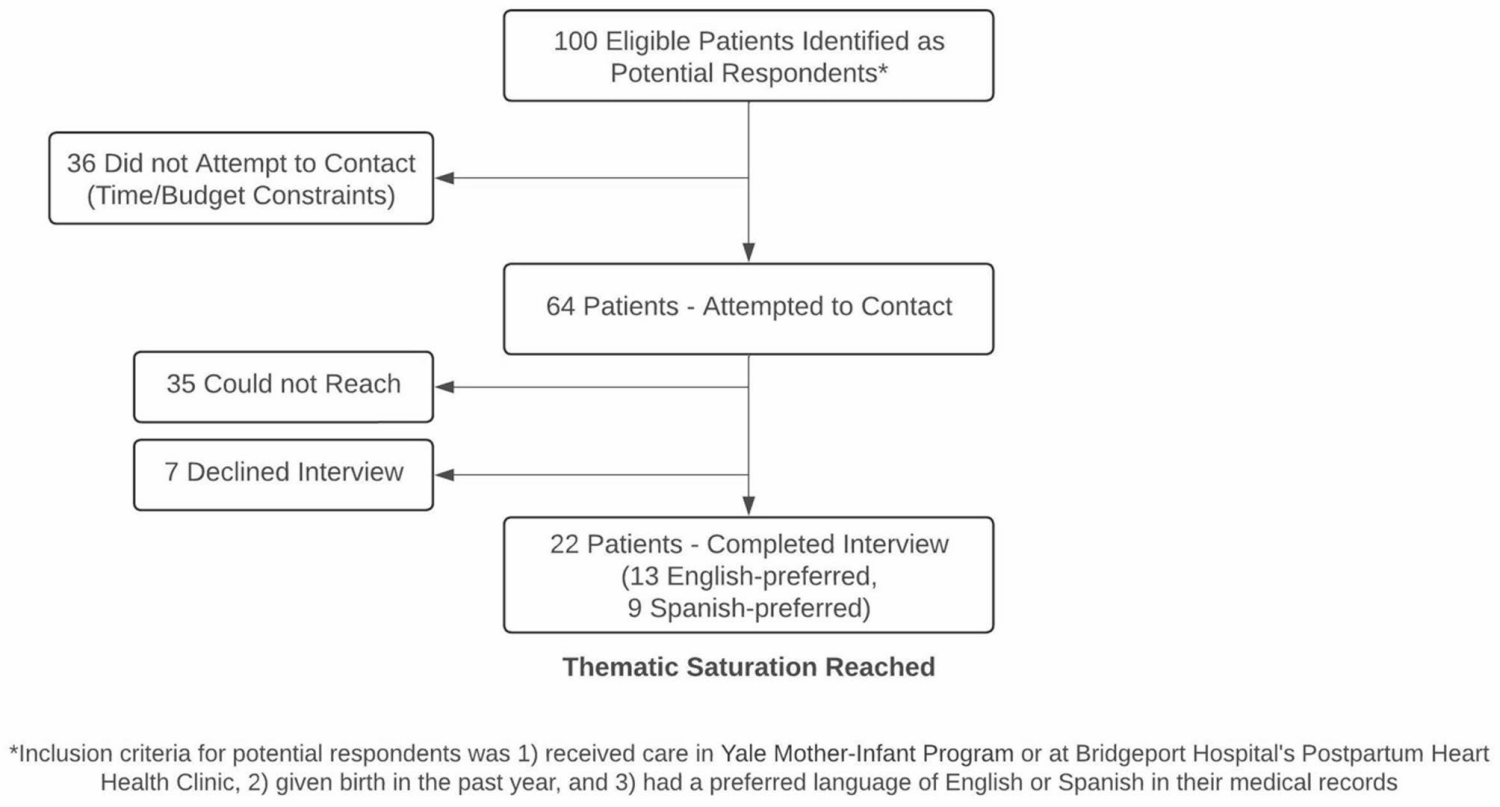
Flow diagram for recruitment and enrollment of mothers who received care at Bridgeport Hospital’s Postpartum Heart Health Clinic or the Yale Mother-Infant Program

Of the 64 eligible patients who received outreach, we had a response rate of 34.4%. Screening and enrollment details are in Fig. 1.

### Sociodemographic characteristics of sample population

Over half (54.5%) of participants identified as Hispanic/Latina, 36.3% identified as non-Hispanic/non-Latina Black, and 31.8% selected ‘I don’t know’ or ‘Other’ for race (Table 1). Over half (59.1%) of participants preferred to speak English in medical settings, while the remaining 40.9% preferred Spanish (Table 1). Participant age at delivery ranged from 18 to 39 years old, and most (72.7%) gave birth to an infant at term (Table 1). Most (81.8%) participants had 4 or more people in their household (Table 1).

**Table 1:** Participant characteristics (*N* = 22)

	*n* (%)
Race/ethnicity of participants	
Hispanic/Latina White	3 (13.6%)
Hispanic/Latina Black	3 (13.6%)
Hispanic/Latina other/don’t know	6 (27.3%)
Non-Hispanic/Non-Latina Black	9 (36.3%)
Non-Hispanic/Non-Latina Other	1 (4.6%)
Participant age at delivery	
18–19	4 (18.2%)
20–24	1 (4.6%)
25–29	4 (18.2%)
30–34	10 (45.5%)
35–39	2 (9.1%)
40+	1 (4.6%)
Household size	
2–3	4 (18.2%)
4–5	11 (50.0%)
6–7	5 (22.7%)
8–9	2 (9.1%)
Infant gestational age at birth	
< 37 weeks	1 (4.6%)
≥ 37 and < 40 weeks	16 (72.7%)
≥ 40 and < 42 weeks	5 (22.7%)
Preferred language in medical settings	
English	13 (59.1%)
Spanish	9 (40.9%)

### Quantitative results

When asked about self-reported health problems during or in the year after pregnancy, half reported blood pressure concerns, 31.8% reported weight concerns, and 18.2% reported diabetes/gestational diabetes (Table 2). Half of the participants stated they had missed a clinic-based appointment due to transportation barriers (Table 2). For participants’ preferences for postpartum care settings, 27.3% preferred the MMC, 22.7% preferred in-home, and 22.7% preferred in-office (Table 2). Although 36.3% of respondents believed they had a risk of health problems in future pregnancies, almost half (40.9%) had not seen a primary care doctor in the past year, and 22.7% stated they did not have a primary care provider. About one-quarter (22.7%) were unsure of future pregnancy health risks (Table 2).

**Table 2:** Patterns of postpartum care receipt

	*n* (%)
Missed an appointment due to transportation problems	
Yes	11 (50.0%)
No	11 (50.0%)
Preference of setting for postpartum care	
Virtual	4 (18.2%)
In-office	5 (22.7%)
In-home	5 (22.7%)
Outside of home in mobile health van	6 (27.3%)
In-person or Virtual	2 (9.1%)
Has a primary care provider	
Yes	16 (72.7%)
No	5 (22.7%)
Not sure	1 (4.5%)
Has seen a primary care provider in the last year	
Yes	13 (59.1)
No	9 (40.9%)
Self-reported health problem during or in year after pregnancy	
Diabetes/gestational diabetes	4 (18.2%)
Blood pressure concerns	11 (50.0%)
Depression	4 (18.2%)
Anxiety	4 (18.2%)
Concerns with weight	7 (31.8%)
Other	4 (18.2%)
Thinks they have a risk of health problems in future pregnancies	
Yes	8 (36.3%)
No	9 (40.9%)
Maybe/not sure	5 (22.7%)

### Qualitative and integrated results

Major themes from the qualitative analysis of interviews included (1) multi-level support centered around mother–infant dyad preference (cross-cutting theme); (2) collective well-being (fostering connection and communal experience); (3) improved provider communication and education (to empower self-care and health knowledge); and (4) responsive and personalized perinatal care (meeting women where they are at). Supplementary quotes can be found in Additional file 5.

### Multi-level support centered around mother–infant dyad preference

Participants identified potential support at multiple socio-ecological levels. On an interpersonal level, participants emphasized the benefit of partners, family, or friends in providing emotional support (checking in and listening to the mother), physical support (helping with household chores or childcare), or financial support (paying for items needed for child).

I hadn’t been working, when I got pregnant, I stopped working. And then when I gave birth, I had a lot of financial and I had a lot of emotional support from my mom and my mother-in-law. They helped me a lot, especially my mom because I had a rough delivery, but my mother-in-law will help me out with cooking and stuff, because I was living with her. My dad will help me out, well my parents will help me out financially sometimes, but mostly it was my man. He was there through it all for real, and I feel like that’s one of the things that he realized, like how big the change was from being not a mom to being a mom having the responsibilities, so I feel like a supportive partner really matters. - Participant #15.

Participants also noted the significance of feeling supported by their health providers and local community and identified helpful healthcare policies during the perinatal period. Common sentiments included the desire to feel cared for and heard by their healthcare providers and to have community networks to lean on for support. They also noted the value of organizational/institutional support, including access to paid parental leave, hospital care navigators, or lactation consultations. Participants often mentioned state or federal programs to support their families through insurance or nutritional programs. Some participants noted the value of nutritional programs such as the Special Supplemental Nutrition Program for Women, Infants, and Children (WIC) and Supplemental Nutrition Assistance Program (SNAP), discussing the programs’ impact on their health and financial stress. However, participant responses reflected potential confusion regarding other programs, as several participants mentioned a lack of adequate information on Medicaid eligibility in Connecticut and access to health services. Some noted that the insurance information gap resulted in high hospital bills and having to enroll in payment plans or financial assistance programs.

When I gave birth, honestly, I did have financial help… right now I have WIC and SNAP. I also have family members who have supported me a lot, like my sister-in-law, brother-in-law, and uncle; they’ve helped me a lot. And at the hospital, the doctors and nurses supported me as well. So, it’s been very, very good help. - Participant #16 (Translated from Spanish).

…They had to repeat an amniocentesis to extract a direct sample from my baby because the results from the first time weren’t conclusive. This created an additional and very intense challenge for me, and I didn’t know that, since I had just found out I was pregnant, I could have applied for aid [insurance], which would have covered all of that and spared me the anguish I felt at the time. It was test after test, bill after bill, and the debt kept growing. So now I have a payment arrangement for those things. If my OBGYN or someone else had told me right when I got pregnant, 'Hey, why don’t you apply for aid?' I would have had less stress during the early stage of my pregnancy. - Participant #9 (Translated from Spanish).

Participants, many of whom had missed visits because of transportation problems (Table 2), also discussed experiences of feeling unsupported in the perinatal period and how the lack of supportive relationships impacted their mental health. They reflected on disconnects with family members and friends who were not understanding or empathetic to their emotional or physical health after giving birth.

I learned that it’s not easy, it’s really life changing. You can’t sleep when you want anymore. You can’t do what you want anymore. And you have to make sure if you do have to handle anything important, you have the support that you need because without no support girl, you’re going to go through the depression, all types of sleep problems, and you’re probably gonna lose yourself. So you need all the support you can get because it’s, it’s really different. It’s totally different. - Participant #8.

### Collective well-being—fostering connection and communal experience

Hand in hand with participants expressing the value of support, many noted a desire to feel connection and belonging with the community of mothers around them and to reach out to those who may feel isolated. Participants wanted to both give and receive support or advice to mothers in similar situations, often noting a collective concern for others. They wanted to pass down knowledge or tips to new parents to ease any burden or stress they could, empathizing with the increased responsibilities and potential hardships of parenting.

I feel like women should give other women advice like say my child’s six months, and then there’s somebody in the office that’s pregnant, that six months lady would talk to the person that’s pregnant, like, ‘Hey, this is what’s gonna happen it might be a little scary,’ give them what’s going on, so they could be more aware Mom to Mom… - Participant #14.

Many participants also expressed an interest in support groups during the postpartum period, in virtual or in-person formats. They described a desire for an established, judgment-free space to express feelings, ask for advice, or share experiences with other mothers, forming a sort of ‘sisterhood’ or ‘kinship.’

[When asked their thoughts on joining a group of women who went through similar things] That’s great if you have the time to do it. Absolutely it helps… A day for brunch during lunchtime. You go. You are able to bring your baby. You’re not able to bring them everywhere. But you know you’re able to bring your baby. No one judging you there, because everyone’s going through the same thing. Everyone shares their experience. And different ways of getting around, things that you didn’t know. - Participant #2.

### Improving provider communication and empowering patients

A common discussion among participants, many of whom had not recently accessed primary care services (Table 2), was the need and desire for improved provider communication and education for patients. They mentioned a preference for genuine and empathetic relationships with their providers, noting previous difficult experiences where providers seemed disinterested or distracted. Participants also reflected on past medical experiences where they felt there was a lack of trust or respect between the provider and the patient.

My mental health was affected pretty substantially because I kept trying to explain to them the problems that were going on…and they kept sending me home…I had preeclampsia during my pregnancy and they still kept sending me home in pain, swollen, could barely move, could barely walk, and it was like, ‘Oh, no, you’re fine’… It was like because they seen it before or seen the same with somebody else they just automatically wrote me off every time…They were so used to other people having same issues like ‘Oh, that’s normal’…Like no it’s not my normal. I’m telling you, it’s not how I usually feel and they kept writing me off, so I ended up having to stay in hospital. - Participant #3.

Some participants who described gaps in care connected these experiences with feelings of disempowerment. They discussed instances where they felt they did not receive enough information about their or their child’s health and commonly expressed a need for further clarity or discussion about their individual pregnancy and postpartum journey and for increased contact with their provider. Many participants mentioned a desire for a more honest and realistic picture of what to expect in childbirth and postpartum recovery, as multiple participants mentioned various health problems including high blood pressure conditions, depression, anxiety, or concerns about weight (Table 2).

I think it would be good for them to provide some guidance. Guidance before giving birth, because when you’re a first-time mom, for example, and you haven’t had babies before, you have so many unanswered questions. And you’d want for someone to explain it to you. So, for first-time moms, it would be great if they gave them guidance about the process of labor, postpartum, whether it will be a C-section or not, just to give them some orientation so that when they’re going through the process, they at least have a little bit of knowledge. - Participant #12 (Translated from Spanish).

Additionally, some participants noted that providers often paid more attention to the child than the mother and wished for more time devoted to focus on maternal health and well-being. Participants spoke of a longing to leave their healthcare appointments feeling heard, educated, and empowered, a sentiment especially relevant as they often mentioned feeling a risk of health problems in future pregnancies (Table 2).

Yeah, I feel like they should keep us as informed as possible and follow up. I wouldn’t say that my doctors did this, but it felt a little bit like that sometimes, when I would go to the appointment, she would ask me questions about if I have anything to tell her and I would go over there to do what I have to do, follow up for those things, make sure that I’m good and that’s it. And not inform you of stuff that can happen or how I felt or any of that, it was just I’m going to get my postpartum checkup and that’s it. And I feel like wouldn’t say care a little bit more, but make sure that we feel comfortable, you know. - Participant #15.

### Meeting women where they are: responsive and personalized perinatal care

The final theme reflects participants expressing a desire to have their health providers recognize and respond to their specific maternal needs. This idea of having specific and person-centered perinatal care builds on the previous theme of individual provider communication, reflecting participants’ preferences on a larger scale within the healthcare system. Participants discussed the idea of providers coming to them, expressing a preference for home and mobile visits (Table 2). Many participants who experienced the MMC emphasized the ease of this type of care delivery and previous positive experiences, indicating a proclivity towards mobile care.

The one support that I got with my second pregnancy, that was really helpful was they allow the, I think it’s like an emergency nurse [APRN], in the ambulance to come to my home instead of me going to the office but I think it was a one-time thing. But I thought that was really helpful, because she brought pampers, machines to check the baby’s vitals. - Participant #19.

Participants also discussed specific services that have or would have been helpful during pregnancy and postpartum, including access to doulas, diaper/food delivery, lactation guidance, and additional mental health resources. Participants noted the value of these services when they were provided or offered to them. Participants also mentioned specific obstetric or pediatric services that would have been helpful.

I was struggling, because my first child latched and my second one didn’t. [It was] a stressful situation and then from one kid being breastfed to the other being formula… It takes a lot for your mental health, so maybe like a lactation or someone that’s going to be there and that helps you, that comes into the house and OK, maybe you guys do it this way, maybe she doesn’t want it and maybe you should pump. Maybe you could try a bottle, like help you in that way… I would say that’s the main point, because a lot of women just be quiet about that kind of thing. - Participant #14.

I think they should be more open to sharing and assisting on how to get stuff like therapy. I know I was offered for them to write a referral, but the therapist they wrote the referral for was just overbooked, you know? I think that’s where the more resources come in. I think they should have more therapists and try to make the process as easy as possible. If I’m telling you I’m depressed, I shouldn’t have to wait a month or two to speak to somebody about that. Stuff like that should be kind of attended to immediately. - Participant #21.

## Discussion

In this mixed-methods study of perinatal care preferences, perspectives, and health knowledge delivery from women who identify with historically underrepresented groups, participants demonstrated a need for multilevel support with specific attention to community and relationship strengthening, provider engagement, and personalized perinatal care. Many had missed postpartum appointments and had not established primary care providers despite reported health concerns, including postpartum hypertension. Participants described preferences and potential solutions for care, including MMCs, with overarching themes noting the importance of multi-level support. Community and connection were solutions mentioned by participants to overcome potential isolation in the postpartum period. Additionally, participants expressed a need for improved communication from medical providers, specifying a desire for respectful provision of health education that empowers and increases patient health knowledge. Participants valued healthcare services that responded directly to the voiced needs of mothers in the postpartum period. Across themes, findings mirrored principles of trauma-informed care, reflecting the need for supportive environments that promote safety, empowerment, and respect for families who may have experienced or been exposed to trauma, violence, or systemic inequities [41–43].

### Clinical and care delivery implications

#### Care delivery preferences

Participants identified structural barriers to care access, particularly related to transportation and childcare, which echoes known barriers to visit attendance for patients from low-income backgrounds [26, 44]. Flexibility in care delivery emerged as one potential solution to the structural barriers experienced by participants. MMCs are uniquely positioned to provide flexible care delivery adjusted to individual circumstances, with adaptable visit frequency, expanded supportive services, and alternative care settings that are convenient and accessible [22, 45, 46]. Frequently, participants conveyed needs that encompassed more than just medical care—including mental health support, breastfeeding consultations, or social services advocacy, as well as access to essential supplies such as diapers and food. These expressed needs align with the capabilities of MMCs and connect to our finding that half of our participants preferred an MMC-style or home-based delivery of postpartum care. Dyadic MMCs have high postpartum attendance: Boston’s “Curbside Care for Moms and Babies” and Yale’s “Mother-Infant-Program” demonstrated 97% and 96% show rates, respectively [7, 21, 47], compared with national postpartum attendance rates of 72.1% [7]. Wider implementation of MMCs can ensure broader access and support for individuals throughout the continuum of care and could expand even further across the life course. Models including the March of Dimes Mom & Baby Mobile Health Centers also provide reproductive and prenatal care [48].

Participants valued provider-patient relationships, expressing that a genuine connection with their medical providers fostered respect and meaningful communication. This aligns with a recent review on perinatal experiences of Black women in the US, which found that positive care experiences included collaborative patient-provider interactions, continuity of care, and culturally centered care [49]. Moreover, provider training to recognize unconscious bias can enhance continuity of care and strengthen patient-provider communication [50]. Many study participants described past negative experiences that eroded their trust in the health system. Such experiences are common; one in six women report experiencing at least one form of mistreatment during childbirth, including a lack of empathy and informed consent when communicating with providers [51–53], with mistreatment occurring more often for women of color or for those who prefer languages other than English [51, 52, 54]. Experiences of perceived discrimination and low provider responsiveness during intrapartum care are associated with increased odds of postpartum visit nonattendance and forgoing postpartum visits [10, 55]. For women from historically marginalized and underrepresented groups, prioritizing accessible, culturally centered, continuous care may mitigate experiences of mistreatment and discrimination through improved provider communication and potentially increase visit attendance.

#### Health education

Study participants also expressed a desire for increased knowledge of specific health-related concepts, including potential postpartum complications, perinatal health conditions, breastfeeding advice, and physical changes. Broadly, our participants expressed a desire to receive a realistic picture of pregnancy, childbirth, and the postpartum period, and they described feeling unprepared for the health consequences during this period, consistent with recent research [56–58]. This highlights the need for clear, accessible information—through phone calls, text messages, or additional in-person visits—to facilitate involvement in care decisions, address concerns in a timely manner, and provide patient-centered support. Women reported feeling insufficiently informed about the postpartum period, and this period is also the most common phase of the prenatal-birth-postpartum continuum for feelings of neglect [54, 56]. A possible reason for this experience of misinformation and neglect is that 45% of women reported refraining from asking questions or expressing concerns during their maternity care [51]. Contributing factors included not wanting to “make a big deal,” being embarrassed, concerns over being perceived as difficult, or feeling rushed through clinical visits [51]. Participants in this study desired to engage in healthcare, reflecting a critical opportunity to improve the patient-provider relationship and empower women to reclaim autonomy. Because empowerment in the perinatal period is associated with improved maternal mental health and infant outcomes [59], trauma-informed, patient-centered, risk-guided care is essential to increase empowerment in the postpartum period [41, 42, 60].

#### Primary care

Establishment and maintenance of primary care during the postpartum period is often low, despite 63% of pregnancy-related deaths occurring after delivery [3]. There is a significant need to address long-term cardiovascular and postpartum health, especially for women in historically marginalized or underrepresented populations, or for those who experience insurance loss [61, 62]. Participants expressed a desire for primary care involvement in both pregnancy and postpartum care but described a disconnect from primary care providers (PCP) during pregnancy. While some participants reported seeing their PCP at some point in the past year, many did not (40.9%), highlighting a gap in the uptake and delivery of preventative care. This finding is backed by existing literature, which indicates that individuals with a recent pregnancy are not more likely to engage with preventative health services compared with those without a recent pregnancy [63]. The postpartum period can serve as a natural segue to ongoing primary care, a crucial approach for preventing and addressing both acute and long-term health needs of women. Complications associated with the perinatal period are often recognized following delivery, underscoring the importance of frequent clinical touchpoints during this critical time frame [6]. The complex interplay of factors contributing to maternal mortality demonstrates that “maternal healthcare” is not and should not be considered solely the responsibility of obstetrics [64, 65].

With irregular primary and missed obstetric care, healthcare delivery during the perinatal period remains fragmented, reflecting a wider concern about the importance of coordinated, multidisciplinary care for optimal maternal health outcomes [26], particularly for individuals experiencing pregnancy complications, such as hypertensive disorders of pregnancy and gestational diabetes mellitus [26, 64]. During the first 12 weeks after delivery, often referred to as the fourth trimester, MMCs can support women to complete or enhance postpartum care and connect with primary care services. MMCs have the potential to serve as one part of a reimagined postpartum healthcare team to reduce long-term health risks and improve maternal health outcomes.

#### Family, peer, and community support

In addition to PCP support, multi-level support networks emerged as key health priorities for participants. They highlighted the essential role of friends and family who can provide interpersonal support for physical and mental health, including taking care of the baby, completing household chores, or being emotionally present and understanding for the participant [56, 66]. Our participants described that support from partners, family, or friends can promote emotional recovery, reinforcing social support as a protective factor against postpartum mental health conditions [66–69].

Many participants expressed interest in fostering community by joining support groups to connect with others with similar experiences, exchange advice, express themselves, and find meaningful connections. Our results reflect existing literature demonstrating a need for postpartum people to share their perinatal experiences, specifically to compare with other mothers and find reassurance [56]. Group prenatal care is one successful model that facilitates discussions to improve education and provide opportunities for social support, endorsed by ACOG due to its high satisfaction and comparable obstetric outcomes [70, 71]. Peer-level postpartum support has been found to successfully mitigate and improve perinatal depression compared to control groups, with both in-person and remote interventions (via telephone or internet) [72, 73]. Our study underscores the need for greater availability of support groups, including in-person and virtual formats. Additionally, it highlights the importance of provider advocacy in familiarizing themselves with community resources and in recommending these resources to patients.

### Policy implications

One potential avenue to increase family support is through paid family and medical leave (FMLA). FMLA policies provide financial support and job protection, providing crucial time for families to bond with their infants and adjust to postpartum life without the pressure of immediate workforce reentry. The absence of universal paid leave in the U.S. disproportionately impacts low-income families, increasing financial strain and forcing many to return to work prematurely after childbirth, often at the expense of their physical and mental health. Early return to work affects both members of the dyad, impacting maternal-infant bonding and lactation. States with paid family leave policies had higher rates of any/exclusive breastfeeding and lower rates of postpartum depression [74, 75].

Societal and policy-level influences play crucial roles in shaping the well-being of women during the perinatal period, with direct impacts on access to healthcare, financial stability, and essential resources. There was variability in participant understanding of their eligibility for public health insurance, and many noted a lack of easy-to-understand insurance information or were unaware of recent state-level eligibility expansions. This knowledge gap can lead to various structural challenges, including delays in obtaining or activating pregnancy-related public health insurance, difficulty finding providers who accept public insurance, and a lack of continuity in care, all of which significantly affect access to services [76]. Expansion of public health insurance in the US has been associated with greater postpartum healthcare utilization and better health outcomes, including lower maternal mortality rates [77, 78]. Beyond emphasizing the need for expanded public health insurance coverage in all states, those that have already implemented this expansion must ensure that patients clearly understand their eligibility criteria and the enrollment process. Insurance coverage dictates the availability of crucial postpartum services, including lactation support, mental health care, and necessary medical visits, yet gaps in coverage and difficulties navigating insurance enrollment create barriers to care.

### Limitations

Limitations include a restricted English- and Spanish-speaking sample in one U.S. state, so findings may not reflect concerns of mothers living in other regions. However, the qualitative analysis did result in thematic saturation [36]. There also may have been a sampling bias, as we were unable to contact all potential participants due to time and budget restraints, as well as difficulty reaching some eligible participants by phone. Participants may have experienced social desirability bias, even though interviews were conducted by researchers not involved in clinical care. Patients who declined participation may have had additional insight but to maintain trust with well-established clinics, we did not pursue further. Lastly, this study was in English and Spanish, which excluded the perspectives of potential participants who preferred other languages. Despite these limitations, the multilingual, diverse perspectives from historically underrepresented communities provided in this mixed-methods study add valuable insights to improve perinatal care.

## Conclusions

In this mixed-methods study of perinatal care preferences and perspectives, we found a critical need for patient-centered, postpartum care that prioritizes accessibility, health education, multilevel support, and genuine provider engagement. Participants emphasized the importance of interpersonal, community, and healthcare system-based support to navigate challenges during the postpartum period. Barriers such as transportation and limited access to primary care contributed to fragmented appointment attendance, reinforcing the need for alternative care delivery models like mobile medical clinics, which were preferred by many participants. Additionally, participants expressed a strong desire for improved provider communication, respectful health education, and more integrated, responsive, and personalized healthcare services that acknowledge the needs of both mothers and their infants. These insights highlight the necessity of expanding innovative, holistic care models that reduce health disparities and enhance postpartum health outcomes for historically marginalized populations.

## Supplementary Information

Below is the link to the electronic supplementary material.

Supplementary Material 1

## Data Availability

The data collected and analyzed during the current study are not publicly available to preserve participant confidentiality, but deidentified data are available from the corresponding author on reasonable request.

## Abbreviations

ACOG: The American College of Obstetrics and Gynecology
FMLA: Family Medical Leave Act
MMC: Mobile Medical Clinic
PCP: Primary Care Provider
NIMHD: National Institute on Minority Health and Health Disparities
WIC: Special Supplemental Nutrition Program for Women, Infants, and Children
SNAP: Supplemental Nutrition Assistance Program
