# Supplementary material for: Perinatal healthcare access, perceived quality, and preferences among historically underrepresented mother–infant dyads: a mixed methods study
Source: BMC Pregnancy Childbirth. 2025 Aug 26;25:887. doi: 10.1186/s12884-025-08029-6 (PMC12379449; doi:10.1186/s12884-025-08029-6)
Supplement: Supplementary file 1 — Supplementary Material 1 [file 12884_2025_8029_MOESM1_ESM.docx]

**Supplementary Materials**

**Additional file 1. Full Interview Guide & Survey**

**Qualitative Interview Guide**

1. **Visit Delivery.**
   1. We are interested in new ideas about health care after childbirth. We know there are lots of medical appointments after leaving the hospital for you and the baby. If you can imagine the perfect way to get health care after birth, what would that look like?
      1. *(Prompt if needed): For example, can you share ideas about when, where, how often, and by whom you would like to be seen?*
2. **Mental health.**
   1. Can you share how your mental health was affected, if at all, around pregnancy and birth?
      1. *(Prompt if needed): For example, did you notice that you felt happier, sadder, or more anxious?*
   2. Can you share what, if anything, was asked about mental health during your pregnancy and after birth?
      1. *(If yes), Who asked about your mental health?*
   3. When a woman is going to have a baby, she may need different kinds of help to stay healthy and feel good. What do you think doctors and nurses could do to help moms during and after pregnancy?
      1. *(Prompt if needed) For instance, do you think doctor visits, support groups, medicines, or other services would be useful?*
      2. *(Second prompt if needed): When a woman has anxiety (nervous) or depression (sad), we usually refer them to a therapist or to a doctor who can prescribe medication. What else could we do?*
3. **Support System.**
   1. Tell me about what help you had during and after your pregnancy.
      1. *(Prompt if needed):* *This can include financial, medical, housing, educational, physical, or emotional support.*
      2. *(Prompt if needed): This can include family, friends, acquaintances, healthcare providers, community networks, or other connections.*
   2. How do you think healthcare providers can help women during and after pregnancy?
      1. *(Prompt if needed): We asked about mental health support, but we are looking for ideas about how we can improve care more generally.*
      2. *(Prompt if needed): Other than your healthcare providers, who else can help women during and after birth?*
   3. What services would help support women during pregnancy and after birth?
      1. *(Prompt if needed):* For example, lactation services, patient care navigators, doulas, social work services
   4. What do you think about joining a group or having chances to talk with other women who went through similar things in pregnancy?
      1. *(Prompt if needed): What would you want that to look like?*
4. **Household Structure.**
   1. We want to know about family household structures, especially when it comes to new mothers and their babies. Can you tell us about the people and relationships in your home?
   2. How many adults, including you, currently live in your home? What are their relationships to you?
   3. Including your newborn, how many children live in your home and what are their ages?
5. **Knowledge.**
   1. We are trying to understand about what women have learned and would like to learn about their health. Please share what you have heard, if anything, about how a woman’s health can change during and after pregnancy.
   2. Tell us about something new you learned about women's health when you were pregnant.
   3. We are interested in sources of health information. Where do you get most of your health information from?
   4. Tell us if you found any new sources of information since you were pregnant?
   5. What kinds of tools would be helpful for you to track your health after birth?
      1. *(Prompt if needed*): For example, remote blood pressure monitoring, apps to track symptoms
   6. What do you wish people talked about while you were pregnant or after having a baby?
   7. Did the doctors find any health issues during your pregnancy that needed more attention or more visits to the hospital?
      1. *(If yes):* If so, what is your understanding of how those conditions affect your health now?
      2. *(If yes):* What is your understanding of how those conditions might affect you later in your life?
      3. *(If yes):* What do you remember being advised about how to follow up on those conditions throughout your life?
6. **Perspectives about Health.**
   1. We want to understand what mothers think it means to be healthy after birth. Can you describe what that means to you?
      1. *(Prompt if needed):* For example, some things people might think about are physical health, mental health, eating well, exercising, getting enough sleep, managing stress, quitting smoking, and breastfeeding. Do any of these things come to mind for you?
7. **Primary Care.**
   1. What do you think the role of a primary care doctor should be during pregnancy and after birth? *Note: consider clarifying and explaining primary care vs OB provider if questioned or answer is unclear, please document if this occurs in the memo section*
   2. Can you share the last time you’ve seen a primary care doctor?
      1. *(If you’ve seen a primary care doctor since you’ve given birth):* Can you share what, if anything, your primary care doctor discussed about your health after birth?
8. **Barriers**
   1. What are some challenges and barriers that you have faced when trying to take care of yourself after birth?
      1. *(Prompt, if needed):* For example, did you have trouble with scheduling appointments, getting to them, finding someone to take care of your child, or dealing with work?
   2. Can you share an example, if any, of a time when you felt you had to choose your baby’s health over your health?
9. **Traditional Practices.**
   1. We want to learn about things we may not know that are important to you, your family, and your community. Please share what else is important for you about your health. This could include traditions or customs.
      1. *(Prompt if needed): Describe what healthy pregnancy means to you.*
      2. *(Prompt if needed): How does a person show care for a new mother?*

**Quantitative Questions**

Was verbal informed consent completed?

- 1. Yes
  2. No

1. Epic Demographic Questions
   1. Age of mother
   2. Gestational age OR How old when delivered?
2. Interview Demographics
   1. Ethnicity: Do you consider yourself to be Hispanic, Latina, or of Spanish origin?
   2. What race or races do you consider yourself to be?
      1. American Indian or Alaska Native, Asian, Black or African American, Native Hawaiian or Pacific Islander, White, Other, Don’t Know, Refused to answer
         1. Inclusive, allow multi-select, allow “don’t know” allow “prefer not to respond”
3. Which would you prefer for your postpartum care?
   1. Virtual
   2. In office
   3. In home
   4. Outside of home in mobile health van
   5. Other
   6. None
4. Have you had any of the following problems during or in the year after your pregnancy?
   1. Blood pressure problems
   2. Diabetes / Gestational Diabetes
   3. Concerns with weight or baby weight
   4. Depression
   5. Anxiety
5. Do you have a primary care doctor?
   1. Yes
   2. No
   3. Not sure
6. Have you seen a primary care doctor within the last year?
   1. Yes
   2. No
   3. Not sure
7. Do you think you have any risk of health problems in future pregnancies?
   1. Yes
   2. No
   3. Maybe
   4. Unsure
8. One of our next steps is to bring together women from the community to provide advice and thoughts about health care for women after childbirth. Would you be interested in more information about this?
   1. Yes
   2. No
9. **Language:** What language do you prefer to speak when you come to the medical center? (allow multi-select)
   1. English
   2. Spanish
   3. Other
10. What language do you feel most comfortable speaking? (allow multi-select)
    1. English
    2. Spanish
    3. Other
11. Have you ever missed a doctor’s appointment because of transportation problems?
    1. Yes
    2. No
12. How would you like to receive the gift card?
    1. Email
    2. Mail
13. How many adults live in the home? *To fill in after interview based on household structure question
14. How many children live in the home? *To fill in after interview based on household structure question

**Additional file 2. COREQ Checklist**

[https://onlinelibrary.wiley.com/pb-assets/assets/17416612/COREQ_Checklist-1556513515737.pdf](https://nam12.safelinks.protection.outlook.com/?url=https%3A%2F%2Fonlinelibrary.wiley.com%2Fpb-assets%2Fassets%2F17416612%2FCOREQ_Checklist-1556513515737.pdf&data=05%7C02%7Cnicole.amodio%40yale.edu%7C0ceb0d4ccddd495966ae08dd8e2ad8ce%7Cdd8cbebb21394df8b4114e3e87abeb5c%7C0%7C0%7C638823036866073305%7CUnknown%7CTWFpbGZsb3d8eyJFbXB0eU1hcGkiOnRydWUsIlYiOiIwLjAuMDAwMCIsIlAiOiJXaW4zMiIsIkFOIjoiTWFpbCIsIldUIjoyfQ%3D%3D%7C0%7C%7C%7C&sdata=yIZX1dpVQg2JljaT38cwxR3iXXY%2B9%2FVD2k2znpmHBMA%3D&reserved=0)

**Additional file 3. STROBE Checklist**

[https://www.strobe-statement.org/checklists/](https://nam12.safelinks.protection.outlook.com/?url=https%3A%2F%2Fwww.strobe-statement.org%2Fchecklists%2F&data=05%7C02%7Cnicole.amodio%40yale.edu%7C0ceb0d4ccddd495966ae08dd8e2ad8ce%7Cdd8cbebb21394df8b4114e3e87abeb5c%7C0%7C0%7C638823036866094548%7CUnknown%7CTWFpbGZsb3d8eyJFbXB0eU1hcGkiOnRydWUsIlYiOiIwLjAuMDAwMCIsIlAiOiJXaW4zMiIsIkFOIjoiTWFpbCIsIldUIjoyfQ%3D%3D%7C0%7C%7C%7C&sdata=1McqYb3FQQorIECvIJG9i%2FnGbmkZlwmpnpioUK3kBVY%3D&reserved=0)

**Additional file 4. Original Spanish Quotations**

| *“Sí, cuando yo me alivié, pues sí, sinceramente tuve ayuda financiera. Eh, hubo una persona que se llama ay, no me acuerdo cómo se llama la persona, pero ella fue ahí al hospital y ella me dijo que ella le podría ayudar checándome la presión cada mes y así... También financieramente bueno, ahorita tengo lo que el WIC y SNAP. Igual tengo familia que me han apoyado mucho, como mi cuñada, mi cuñado, mi tío me han apoyado bastante y este y en el hospital, pues igual me apoyaban los doctores, las enfermeras, así bueno, ayuda muy, muy buena.” - Participant #16*  "Yes, when I gave birth, well, honestly, I did have financial help. There was a person, I can’t remember her name, but she came to the hospital and told me that she could help me by checking my blood pressure every month and so on. Also, financially, right now I have WIC and SNAP. I also have family members who have supported me a lot, like my sister-in-law, brother-in-law, and uncle; they’ve helped me a lot. And at the hospital, the doctors and nurses supported me as well. So, it’s been very, very good help." *Participant #16 (Translated from Spanish)* |
| --- |
| *“...me repitieron un día una amniocentesis para sacar una masa directa de mi bebé porque no eran concluyentes que me hicieron la primera vez. Entonces esta genero un giro adicional y muy fuerte para mí y después no sabía que apenas yo estaba embarazada, podía haber aplicado para aid (seguro de salud) que me hubiera cubierto todo ese tema y me hubiera sentido toda la toda la angustia que que sentía en ese momento, puesto que era examen tras examen incluía, factura tras factura y se iba incrementando una deuda. Entonces yo ahora tengo un convenio de pago por estos por esas cosas, cuando si mi OB o alguna persona apenas estuve embarazada, dice, oye, por qué no aplicas para aid Hubiera tenido menos estrés en el en la primera etapa de mi de mi embarazo.”*  “...They had to repeat an amniocentesis to extract a direct sample from my baby because the results from the first time weren’t conclusive. This created an additional and very intense challenge for me, and I didn’t know that, since I had just found out I was pregnant, I could have applied for aid [Insurance], which would have covered all of that and spared me the anguish I felt at the time. It was test after test, bill after bill, and the debt kept growing. So now I have a payment arrangement for those things. If my OBGYN or someone else had told me right when I got pregnant, “Hey, why don’t you apply for aid?” I would have had less stress during the early stage of my pregnancy.” - Participant #9 (Translated from a Spanish interview) |
| *“Bueno, yo pienso que sería bueno, que le digan que le den como una orientación. Una orientación antes de dar a luz, porque cuando tú eres, por ejemplo, primeriza que tú no has tenido bebés, sí, tú tienes muchas preguntas. No resueltas y uno quisiera como tú sabes como que uno se lo digan, o sea que a las madres que sean primerizas que le den una orientación de cómo está el proceso del parto del posparto, si será cesaría, si no será Cesárea, que le den como una orientación para que cuando ya ellas estén en el proceso tengan por lo menos un poquito de conocimiento.”*  “I think it would be good for them to provide some guidance. Guidance before giving birth, because when you’re a first-time mom, for example, and you haven’t had babies before, you have so many unanswered questions. And you’d want for someone to explain it to you. So, for first-time moms, it would be great if they gave them guidance about the process of labor, postpartum, whether it will be a C-section or not, just to give them some orientation so that when they’re going through the process, they at least have a little bit of knowledge.” - Participant #12 (Translated from a Spanish interview) |

**Additional file 5. Supplemental Participant Quotes**

| **Theme Name & Description** | **Example Quote** |
| --- | --- |
| **Multilevel Support** – ***Cross-cutting theme*** *Importance of feeling supported by family, friends, healthcare providers, hospital systems, and policies.* | *“This is my first child. I don't really know what to expect, but I did recently speak with like a like a case like health case worker and she's been really supportive in getting me help as far as like therapy, food stamps…so I feel like it's been kind of it's been helpful as far as like getting me kind of involved in the community as well as making sure that me and my daughter are taken care of…I feel like this is top tier care…she’s been so helpful..like, you know, showing me all these resources and I definitely want to take advantage of them" - Participant #4*  *"Well, thank God, the doctor I had was very good to me. Like I mentioned, there are doctors who don’t ask patients anything, but the doctors I’ve had have helped me a lot, especially emotionally, because they support you emotionally by giving you words of encouragement, since you come in feeling unwell. Yes, they are very kind." - Participant #6 (Translated from Spanish)*  *"Well, at the moment, I don’t have a primary doctor. As I mentioned, because of my insurance, I wasn’t able to have a primary doctor, and up to now, I still don’t have one because I try to make appointments, but they don’t have availability." - Participant #6 (Translated from Spanish)* |
| **Collective Wellbeing—Fostering Connection and Communal Experience**  *Speaking with other parents to connect or exchange advice and have an established space to do so* | “*I don’t know, maybe opening a workshop for moms who want to come and talk, because that’s not my case, but I’ve met many moms who go through tough situations, would be great. How nice it would be to have an open space for them to de-stress, maybe do things to keep their minds occupied, open workshops, or even just have talks where they can vent*. - Participant #11” (Translated from Spanish)”  *[When asked their thoughts on joining a group of women with similar experiences] I would like that like it would, even if you can't get because it's very hard for transportation with some people who don't drive and, you know, people have a lot of kids or, you know, it's the kids running around. You don't have a babysitter. So I personally think that it would be OK. Or over the phone or and do something where people just talk and exercise, you know, certain things that's going on. Because I mean, a lot of people have a lot of stuff going on, but everybody don't know that because a lot of people hiding very well, like I do. - Participant #5*  *"Yes, it would be really nice to hear the opinions of other people, of other women, and to know what solutions they choose, because sometimes different ideas are helpful to keep moving forward." - Participant #18 (Translated from Spanish).* |
| **Improved Provider Communication and Education to Empower Self-care and Health Knowledge** – *Desire for clarity on medical discussions for patient to advocate for themselves and feel empowered* | *“In regards to that, like there was a lot of questions that I found out about or like I was informed about like after I gave birth, I feel like doctors sometimes try to hide like certain things from the patient and I feel like it's better to let them know instead of like just waiting for something to happen, you know.” - Participant #15*  *“Well, my experience, I didn’t have a doctor who, like, I mean, psychologically, or how can I put it? Yes, I would have liked that when, for example, women give birth, there would be someone to explain things to them. Like, when I had my baby, no one taught me how to bathe him in the hospital, and... So, I didn’t know how to bathe him. Thank God I have my sister-in-law, and she helped me and taught me how to bathe him, but at the hospital, no, there wasn’t anyone, well, no one supported me with that—teaching me how to bathe the baby. I would have also liked if there were someone who, at that moment, could talk to you about the psychological aspect, about what you are going to experience with the baby, what it can be like, what things could happen, and so on." - Participant #16* (Translated from Spanish) |
| **Meeting Women Where They Are At: Responsive and Personalized Perinatal Care**  *Healthcare delivery that is unique to women and prioritizes their needs* | *“I feel like some of these doctors put on, and the nurses like you can kind of tell that they kind of like push their own personal beliefs on you. And I think that can get uncomfortable at times because, like we all have our own perception of how we want our life to go and how we want to raise our own children, you know, and I think you have to be respectful. We may not always agree with somebody’s you know ideas and stuff, but you have to be respectful.” - Participant #19*  *"...But I also like it because, I mean, from the moment we are in the hospital, they are taking care of us, checking in at every moment after the appointments. It's all this attention for me and for my child. And what I liked was that, just like when my child had his check-ups, they came to see me at home, and that was good for me because I didn’t have to go all the way to the clinic where I had to take the baby, they came to see me here. And what I would like is that, for example, during the first few months of my baby’s life, I had some doubts, some concerns about what was happening, but sometimes it was like, I don’t know if it was me not understanding well, or the doctors not explaining it clearly, I don’t know. But yes, I would like that if I tell them, 'Oh, I feel worried about my child in certain things,' that someone, a specialist, would address it so I could feel more reassured."* - Participant #16 (Translated from Spanish)  *“So I actually have perfect, you know, not exactly perfect, but I have perfect health here actually because I have like the drive up, the drive up clinic came because to see my baby so I wouldn't have to leave the house. So it actually worked out for me. They drove up to the house for me.” - Participant #3*  *“They, what doctors should do for females after pregnancy, I feel like, certain doctors could be very hard on the mother. Because you know you're supposed to go and get be seen right after you get out of the hospital and four weeks, six weeks. Sometimes you know, parents don't want to do that. Sometimes they don't want to go to those checkups and they rather have a doctor give them tell them everything that they need so that they can get all the rest they can get and be home with their child because it's enough with us already being in the hospital and not being able to rest. So once you know, mothers leave, they don't want to be coming back tomorrow or three days later but you know, that's something that they recommend. I feel like if they do need to check up, they might as well just start at 2 weeks instead of the next day because that's just crazy. A mother should be able to enjoy her company with her child and then a week or two later, then we know, go see the doctors and see what's going on, how they're doing. You know what I mean? It’s okay for you to call they do suggest doctors coming to the house to check on the kids, but you know what I mean? With the checkups and stuff it should be a little bit more in check with us.” - Participant #8* |
